# Clinical efficacy comparison of internal fixation of locking compression plate and cannulated screw in treatment of elderly femoral neck fractures—a retrospective study

**DOI:** 10.3389/fsurg.2025.1600331

**Published:** 2025-08-25

**Authors:** Fei Sun, Yu Sun, Wei Li, Zhi Tang, Enxu Liu, Zhaoyong Li, Shaofeng Yang

**Affiliations:** ^1^ Department of Orthopedics, Xiangtan Hospital of Traditional Chinese Medicine, Xiangtan, Hunan, China; ^2^ Graduate School of Hunan University of Traditional Chinese Medicine, Yuelu District, Changsha, Hunan, China; ^3^ The First Affiliated Hospital of Hunan University of Chinese Medicine, Yuhua District, Changsha, Hunan, China

**Keywords:** femoral neck fracture, cannulated screw fixation, locking compression plate, early functional recovery, complications

## Abstract

**Objective:**

To explore the clinical efficacy of internal fixation of locking compression plate and Cannulated Screw in treatment of elderly femoral neck fractures.

**Methods:**

175 patients with femoral neck fractures admitted to our hospital from January 2022 to December 2022 were enrolled in the study. 93 cases in the control group were treated with Cannulated Screw internal fixation, and 82 cases in the observation group were treated with locking plate internal fixation. The control group was treated with cannulated screw internal fixation, while the observation group was treated with locking compression plate internal fixation.

**Results:**

Compared with the control group, the observation group had a significantly shorter time for partial weight-bearing exercise, with a statistically significant difference (*p* < 0.05), and a significantly lower incidence of postoperative complications, with a statistically significant difference (*p* < 0.05). The ROM of hip extension-flexion at 1 month and 6 months after operation and the ROM of hip internal rotation-external rotation at 1 month after operation in the observation group were significantly higher than those in the control group, and the differences were statistically significant (*P* < 0.01). The VAS score of the observation group was significantly lower than that of the control group at 1 month after operation, and the difference was statistically significant (*P* < 0.01).

**Conclusion:**

Both locking compression plate internal fixation and cannulated screw internal fixation are effective in the treatment of elderly femoral neck fractures. Compared with cannulated screw internal fixation, locking compression plate internal fixation helps patients to engage in early functional exercise and has a lower incidence of postoperative complications.

## 1 Introduction

Femoral neck fracture, as a common type of fracture in orthopedics, accounts for about 3.6% of all fractures (1) and over 50% of hip fractures (2). This condition is more prevalent in the elderly population and its incidence is increasing annually with the aging population in China (3, 4). Femoral neck fractures have a high rate of disability, and improper treatment can significantly impact a patient's quality of life (5). Currently, treatment methods for femoral neck fractures mainly include conservative treatment, open reduction internal fixation, and joint replacement. Joint replacement allows early mobilization of patients and promotes functional recovery, but it can also lead to serious complications like pulmonary infections, deep vein thrombosis, and even sudden death. When considering joint replacement treatment clinically, patient age and physical condition should be thoroughly evaluated (6, 7). Elderly patients with femoral neck fractures often have concomitant underlying health conditions, making them less tolerant of hip replacement surgery. For non-displaced Garden I and II type fractures, internal fixation is typically the preferred clinical treatment approach. However, there is still significant controversy regarding the specific choice of internal fixation treatment method.

Internal fixation of cannulated screw for the treatment of elderly femoral neck fractures offers such advantages as minimal trauma, simple surgical procedures, and relatively low costs (8, 9). It has been widely used in clinical practice but still has some drawbacks, like the potential to cause femoral neck shortening in patients. This is especially concerning for elderly patients who often have varying degrees of osteoporosis, leading to significant controversy regarding its reliability (10). Locking compression plates have been extensively employed in the treatment of elderly limb fractures, demonstrating good clinical outcomes. However, there are relatively few reports on their use in treating elderly femoral neck fractures.

Therefore, this study aims to investigate the clinical efficacy of locking compression plate vs. cannulated screw with regard to internal fixation in the treatment of elderly femoral neck fractures by collecting data from 175 femoral neck fracture patients admitted to our hospital from January 2022 to December 2022.

### 1.1 Data and research methods

#### 1.1.1 Research objects

175 cases of femoral neck fractures treated at Affiliated Hospital of Hunan University of Traditional Chinese Medicine from January 2022 to December 2022 were selected for this study. The clinical choice between hollow screw fixation and locking compression plate fixation mainly depends on the fracture type, bone quality, and stability requirements. Hollow screws are suitable for simple fractures with good bone quality and no obvious displacement, while locking compression plates are preferred for complex fractures, osteoporosis, or cases requiring greater stability to promote early functional recovery and reduce complications.

#### 1.1.2 Inclusion and exclusion criteria

The inclusion criteria were as follows: (1) Age ≥ 60 years; (2) Unilateral femoral neck fracture diagnosed by imaging; (3) Garden type I or II classification; (4) Informed consent from patients and family members.

The exclusion criteria were as follows:(1) Concurrent other fractures; (2) Concurrent severe cardiovascular or cerebrovascular diseases or mental disorders; (3) Old fractures; (4) Loss to follow-up. Based on the different internal fixation methods, the patients were divided into a control group and an observation group. The control group underwent treatment with internal fixation of cannulated screw, while the observation group received internal fixation of locking compression plate. The control group consisted of 36 males and 57 females, with an average age of (72.83 ± 3.17) years and an average time from injury to surgery of (8.42 ± 7.21) hours. The observation group included 32 males and 50 females, with an average age of (72.65 ± 3.23) years and an average time from injury to surgery of (9.12 ± 7.24) hours. There were no statistically significant differences in gender, age, side of fracture, time from injury to surgery, and underlying comorbidities between the two groups (*p* > 0.05), as shown in Table 1. All patients were treated by the same group of doctors, and informed consent was obtained from all patients.

**Table 1:** Comparison of general data.

Group	n	Gender (Male/Female, cases)	Age (years)	Fracture Side (Left/Right,cases)	Time from Injury to Surgery (h)	Comorbidities (cases)		Pauwels angle		
						Hypertension	Diabetes	Ⅰ	Ⅱ	Ⅲ
Control group	93	36/57	72.83 ± 3.17	41/52	8.42 ± 7.21	14	10	15	52	26
Observation group	82	32/50	72.65 ± 3.23	34/48	9.12 ± 7.24	16	12	13	47	22
χ^2^/*t*-Value		0.01	0.37	0.12	0.64	0.61	0.60	0.78		
*p*-Value		0.97	0.71	0.73	0.52	0.43	0.44	0.67		

**Figure 1:**
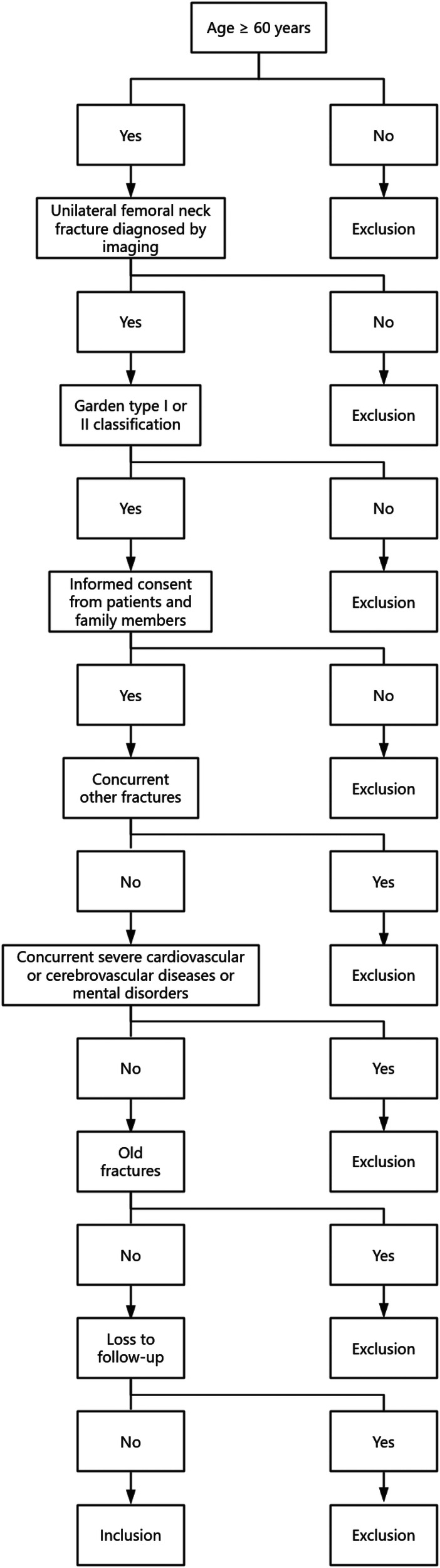
Flow diagram of patient screening and enrollment.

In the clinical evaluation of treatment strategies for elderly patients with femoral neck fractures, clearly defining inclusion and exclusion criteria is essential to ensure the scientific rigor and reproducibility of the study. To guarantee the homogeneity of the sample and the reliability of the data, this study implemented a strict screening process based on patients' demographic characteristics, imaging diagnoses, and clinical conditions. The detailed selection process is visually presented in Figure 1.

Figure 1 illustrates the patient selection flowchart used in this study. Eligible participants had to meet the following criteria: age ≥ 60 years, diagnosis of unilateral femoral neck fracture confirmed by imaging, classification of the fracture as Garden type I or II, and provision of informed consent by the patient and family members. Additional exclusion criteria included the presence of concurrent fractures, severe cardiovascular or cerebrovascular diseases, mental disorders, old fractures, or loss to follow-up. The diagram clearly outlines the decision-making path at each step, showing the “Yes” and “No” branches that determine the patient's eligibility status. The logical and structured design of this flowchart reflects the systematic and rigorous approach to case selection, forming a solid foundation for the subsequent comparative clinical analysis.

**Figure 2:**
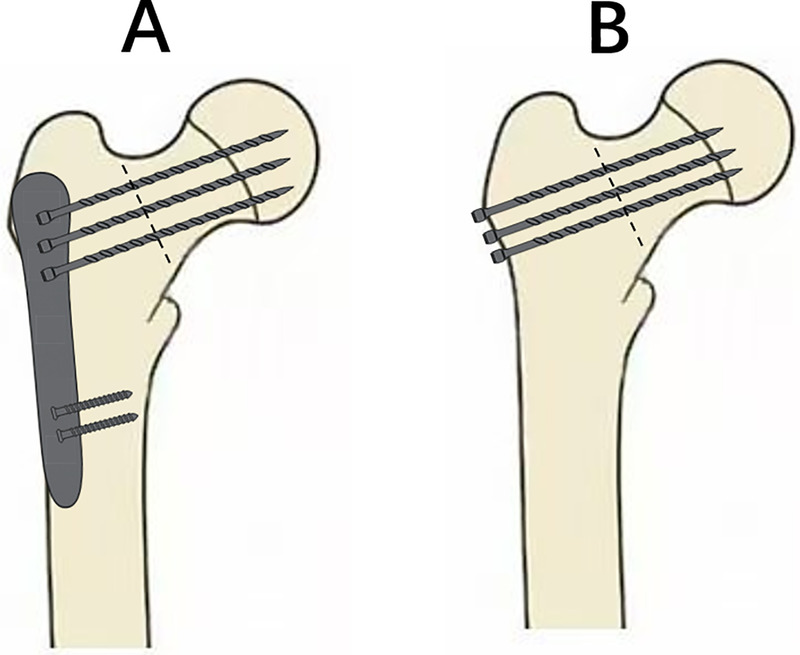
Schematic illustration of internal fixation methods for femoral neck fracture. **(A)** Locking compression plate. **(B)** Cannulated screw fixation.

In evaluating the clinical outcomes of different internal fixation methods for femoral neck fractures in elderly patients, it is important to visually illustrate the structural characteristics of each surgical technique to better understand their biomechanical differences and postoperative performance. Figure 2 provides a schematic comparison of the two surgical approaches employed in this study, highlighting the differences in implant configuration and insertion trajectory between locking compression plate fixation and cannulated screw fixation.

On the left side of Figure 2 is the locking compression plate fixation, where the plate is placed along the lateral cortex of the femur and fixed to the femoral neck and head using multiple locking screws that cross the fracture line, forming a stable multi-point support structure. On the right side is the cannulated screw fixation, in which three parallel screws are arranged in an inverted triangle and inserted obliquely through the fracture line into the femoral head from the lateral cortex, achieving stabilization through interfragmentary compression. These two techniques differ significantly in terms of insertion direction, support mechanism, and biomechanical behavior, which can influence early postoperative weight-bearing and functional recovery.

#### 1.1.3 Surgical procedures

##### 1.1.3.1 Both groups of patients were treated by the same team of experienced doctors

###### 1.1.3.1.1 Control group

The internal fixation of cannulated screw was used in the control group, with general anesthesia administered during the procedure. The patient was placed in a supine position, and three triangular guide wires were inserted into the femoral neck along the lateral aspect of the greater trochanter. The position was confirmed under x-ray fluoroscopy, and a 3.0 cm incision was made at the point where the guide wire entered the skin. Subsequently, a cannulated screw (7.3 mm) of appropriate length was inserted approximately 0.5 cm from the cartilage surface of the femoral head. Layer by layer closure of the incision was performed to complete the surgery. The control group was operated on by the same team of surgeons using the aforementioned method.

###### 1.1.3.1.2 Observation group

**Figure 3:**
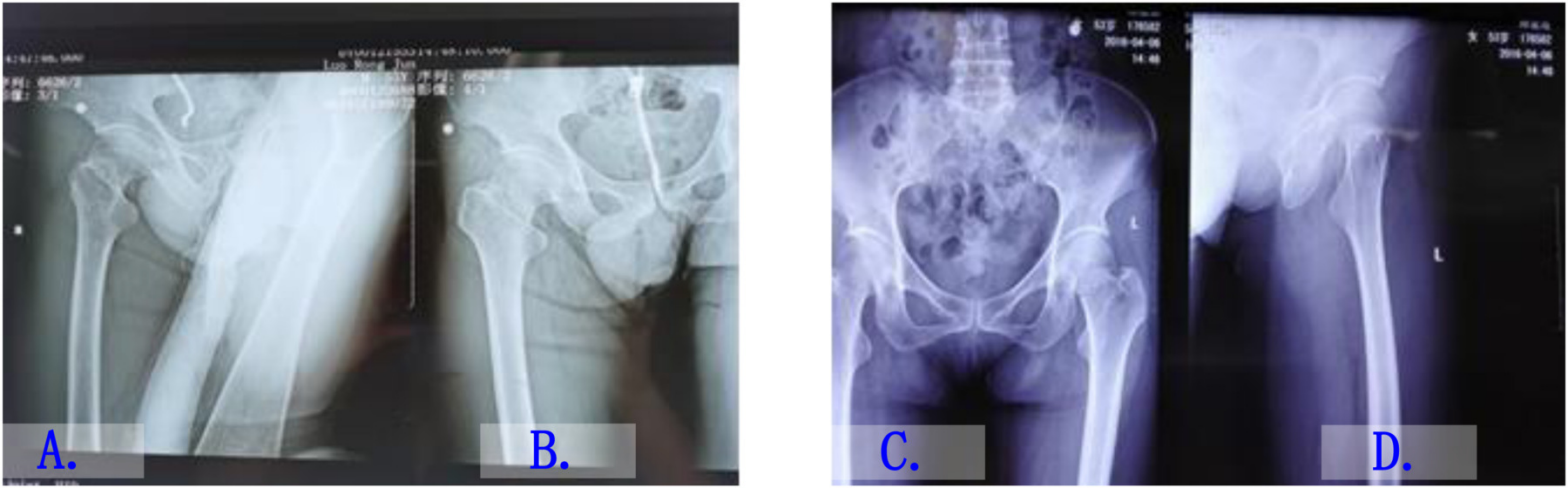
Imaging examination images of typical cases. **(A)** Preoperative x-ray of a patient with a right femoral neck fracture. **(B)** Postoperative x-ray after fixation with a locking compression plate. **(C)** Preoperative x-ray of a patient with a left femoral neck fracture. **(D)** Postoperative x-ray after fixation with cannulated screws.

The internal fixation of locking compression plate was employed in the observation group, with general anesthesia administered during the procedure. The patient was positioned in a supine position with the affected limb fixed on a traction bed. A longitudinal incision of approximately 2 cm was made along the upper edge of the lesser trochanter, and the skin, subcutaneous tissue, iliofemoral ligament, and lateral muscles of the thigh were sequentially separated. Three guide wires were used to fix the femoral neck end under C-arm fluoroscopy. A steel plate was placed against the long axis of the femur under C-arm fluoroscopy, ensuring parallel alignment, and the guide wire was positioned above the femur by at least 3 mm. Using the same steel plate as a reference, a 2 cm incision was made in the corresponding skin, and two distal screws were inserted and fixed to the distal steel plate. Subsequent drilling was performed, followed by the insertion of three cannulated screws into the head and neck. Fluoroscopy was used to confirm the good position of the steel plate and screws. Layer by layer closure of the incision was performed to complete the surgery. All patients were evaluated by imaging and met the surgical conditions. The patients in the observation group were all operated on by the same team of surgeons using the aforementioned method. The imaging examination of typical cases is shown in Figure 3.

The postoperative rehabilitation protocols for the two groups of patients were as follows:On the first postoperative day, a pneumatic compression device was used to prevent deep vein thrombosis, and patients were instructed to perform ankle pump exercises (with both lower limbs extended, dorsiflex the ankle to the maximum angle and tense the calf muscles for 5 s, then plantarflex the ankle to the maximum angle and hold for another 5 s before relaxing) as well as quadriceps contraction training (place a rolled towel under the knee, then contract the quadriceps and hold the contraction for 30 s).

#### 1.1.4 Observational indicators

General Data of Patients in Two Groups: This includes gender, age, fracture side, time from injury to surgery, and underlying comorbidities. (2) Perioperative Data of Patients in Two Groups: This includes surgical duration, incision length, intraoperative blood loss, postoperative decrease in hemoglobin levels, and wound healing status. (3) Weight-Bearing Exercise Duration and Fracture Healing Time in Two Groups of Patients: This data pertains to the time for weight-bearing exercise and fracture healing. Fracture healing was assessed by postoperative follow-up x-rays. (4) Hip Joint Harris Score in Two Groups of Patients (11):The Harris score for the hip joint was evaluated in both groups at 6 months postoperatively, including assessments of function, pain, joint mobility, and deformity. The total score is 100 points, with higher scores indicating better hip joint function. (5) Postoperative Complications in Two Groups of Patients: This includes occurrences of pulmonary infection, urinary tract infection, pressure sores, deep vein thrombosis, femoral neck shortening, nonunion of fractures, and avascular necrosis of the femoral head.

#### 1.1.5 Statistical methods

The data was analyzed with SPSS 21.0 statistical software. The Shapiro–Wilk test was used to determine whether the data followed a normal distribution.Continuous data are presented as “ $\bar{x}\pm s$,” and intergroup comparisons were conducted with *t*-test. Categorical data are presented as cases or percentages, and comparisons between the two groups were performed with χ2 test. *P* < 0.05 was considered statistically significant.

## 2 Results

### 2.1 Comparison of perioperative data between two groups

The surgery duration, incision length, intraoperative blood loss, and postoperative hemoglobin decrease in the control group were (34.42 ± 8.15) min, (3.32 ± 0.30) cm, (73.25 ± 7.89) ml, and (1.11 ± 0.10) g/L, respectively. In the observation group, these values were (37.15 ± 8.23) min, (3.35 ± 0.31) cm, (75.11 ± 8.44) ml, and (1.07 ± 0.14) g/L, respectively.There were no statistically significant differences in surgical duration, incision length, intraoperative blood loss, postoperative decrease in hemoglobin levels, and wound healing grades between the two groups (*p* > 0.05), as shown in Table 2.

**Table 2:** Comparison of perioperative data.

Group	n	Surgical time (min)	Incision length (cm)	Intraoperative blood loss (ml)	Postoperative hemoglobin decrease (g/L)	Postoperative hemoglobin decrease (g/L)		
						A	B	C
Control group	93	34.42 ± 8.15	3.32 ± 0.30	73.25 ± 7.89	1.11 ± 0.10	93	0	0
Observation group	82	37.15 ± 8.23	3.35 ± 0.31	75.11 ± 8.44	1.07 ± 0.14	82	0	0
χ^2^/*t*-Value		2.20	0.65	1.51	2.20	–	–	–
*p*-Value		0.11	0.52	0.13	0.41	ns	–	–

### 2.2 Comparison of weight-bearing exercise time and fracture healing time between two groups

The time to partial weight-bearing exercise and fracture healing time in the control group were (14.85 ± 8.12) days and (5.01 ± 1.11) months, respectively. In the observation group, these times were (3.82 ± 1.31) days and (4.65 ± 1.03) months, respectively. Patients in the observation group had significantly less partial weight-bearing time in comparison to the control group, with a statistically significant difference (*p* < 0.05). There was no statistically significant difference in fracture healing time between the two groups (*p* > 0.05), as shown in Table 3.

**Table 3:** Comparison of weight-bearing exercise and fracture healing time.

Group	n	Partial weight-bearing exercise time (days)	Fracture healing time (months)
Control group	93	14.85 ± 8.12	5.01 ± 1.11
Observation group	82	3.82 ± 1.31	4.65 ± 1.03
*t*-Value	–	12.16	2.21
*p*-Value	–	<0.05	0.20

### 2.3 Comparison of harris scores between two groups

**Figure 4:**
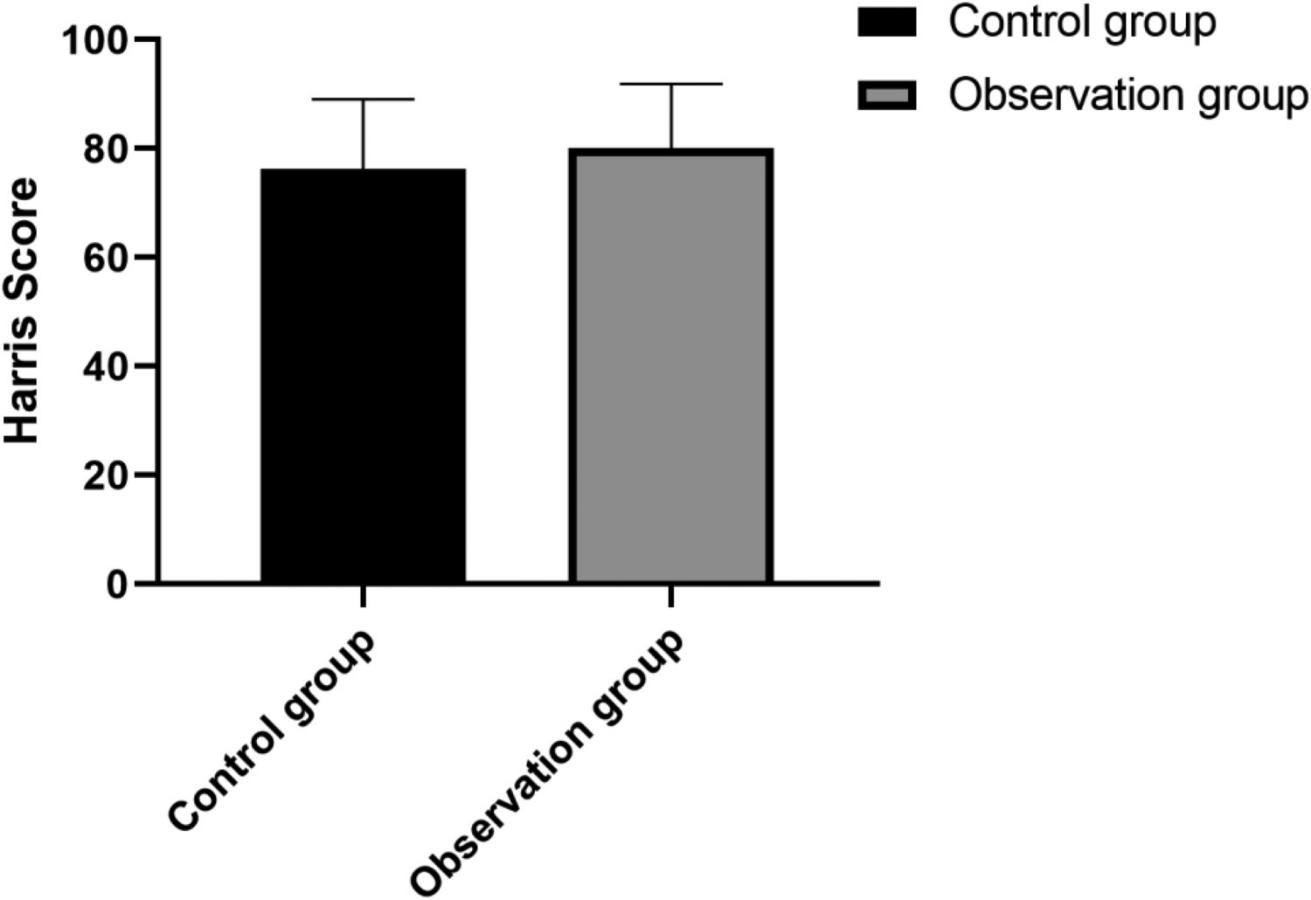
Comparison of Harris scores.

There was no statistically significant difference in Harris scores between the two groups (*p* > 0.05), as shown in Figure 4.

### 2.4 Comparison of postoperative satisfaction between the two groups of patients

There was no statistically significant difference in postoperative satisfaction between the two groups, as shown in Table 4.

**Table 4:** Comparison of postoperative satisfaction between the two groups of patients.

Group	n	Satisfaction (n)	Dissatisfaction (n)	Level of satisfaction (n,%)
Control group	93	90	3	90 (96.8)
Observation group	82	80	2	80 (97.6)
χ2 Value				0.31
*p*-Value				0.76

### 2.5 Comparison of postoperative complications between two groups

The total complication rate was 36.56% in the control group and 15.85% in the observation group. The overall postoperative complication rate in the observation group was significantly lower than that in the control group, with a statistically significant difference (*p* < 0.05), as shown in Table 5.

**Table 5:** Comparison of postoperative complications.

Group	n	Pulmonary infection	Urinary tract infection	Pressure sores	Deep vein thrombosis	Femoral neck shortening	Fracture nonunion	Total complications (n, %)
Control group	93	11	8	2	2	10	1	34（36.56%）
Observation group	82	6	3	0	0	3	0	12（14.63%）
χ^2^ Value								3.29
*p*-value								P<0.05

The ROM of hip extension-flexion at 1 month and 6 months after operation and the ROM of hip internal rotation-external rotation at 1 month after operation in the observation group were significantly higher than those in the control group, and the differences were statistically significant (*P* < 0.01). The VAS score of the observation group was significantly lower than that of the control group at 1 month after operation, and the difference was statistically significant(P < 0.01), as shown in Table 6.

**Table 6:** Comparison of postoperative follow-up data between the two groups.

Group	n	Hip extension-flexion ROM(°)		Internal-external rotation ROM(°)		VAS score	
		One month after operation	Six month after operation	One month after operation	Six month after operation	One month after operation	Six month after operation
Control group	93	106.51 ± 7.23	125.43 ± 7.22	31.14 ± 3.25	47.25 ± 3.43	4.43 ± 0.51	2.12 ± 0.57
Observation group	82	112.21 ± 7.91	132.72 ± 8.22	36.33 ± 4.01	47.39 ± 3.59	3.41 ± 0.40	2.11 ± 0.69
T-Value		4.98	6.23	9.45	0.26	14.58	0.10
*P*-Value		<0.01	<0.01	<0.01	0.79	<0.01	0.92

## 3 Discussion and conclusion

Femoral neck fracture refers to a fracture that occurs between the femoral head and the base of the femoral neck, and is one of the common types of fractures in clinical practice. It can be classified into Garden I to IV types based on the degree of displacement (12, 13). Garden I type indicates an incomplete fracture where the fracture site is not completely separated; Garden II type refers to a complete fracture with complete separation of the fracture site but without displacement; Garden III type refers to a complete fracture with partial displacement at the fracture site; Garden IV type indicates a fracture with complete displacement. Due to its unique anatomical structure and blood supply situation (14), femoral neck fractures pose significant treatment challenges, bringing enormous challenges to clinical work. Current treatment methods mainly include conservative treatment, open reduction internal fixation, and joint replacement. For elderly patients with femoral neck fractures, conservative treatment often involves prolonged bed rest, severely impacting limb function exercise and the patient's quality of life, while increasing the risk of pulmonary infections and deep vein thrombosis (15–17). Joint replacement is primarily used to treat Garden III and Garden IV type femoral neck fractures with displacement (18), and has shown good clinical outcomes.For Garden I and Garden II type femoral neck fractures without displacement, considering the relatively low occurrence rate of avascular necrosis of the femoral head, and the relatively easier fracture healing process, internal fixation surgical treatment is generally chosen (19, 20). Commonly used internal fixation methods in clinical practice include closed reduction percutaneous techniques or minimally invasive approaches, where the internal fixation material is inserted into the affected limb. This method avoids opening the hip joint capsule, which can better preserve blood flow to the femoral head (21), providing favorable conditions for postoperative limb function recovery. However, there is still significant controversy regarding the selection of internal fixation materials and techniques.

Geriatric femoral neck fracture is a common orthopedic condition that often involves varying degrees of bone loss or osteoporosis due to the advanced age of the patients (22, 23).This renders the bones of elderly patients exceptionally fragile, significantly increasing the risk of secondary injury during surgery. To ensure the safety and efficacy of the surgery, selecting the appropriate internal fixation method is crucial. Improper internal fixation methods may not only affect the healing of the fracture but also lead to serious postoperative complications (24).Currently, cannulated screw fixation has been widely used in the treatment of non-displaced Garden I and Garden II type femoral neck fractures. This method is advantageous due to its low cost, simplicity, high safety, and minimal surgical trauma. However, there are some drawbacks associated with this technique, such as suboptimal fracture reduction postoperatively and fixation loosening, which can impact patient recovery and prognosis. In recent years, locking compression plate fixation technology has garnered attention in clinical practice (25, 26). In contrast to cannulated screw fixation, locking compression plate fixation combines cannulated screws in the femoral neck with a steel plate in the femoral shaft, forming a stable overall framework that provides higher strength and stability (27–29). This structure can improve fracture healing and reduce the risk of fixation loosening. Although locking compression plate fixation theoretically offers certain advantages, there is still some controversy regarding its treatment outcomes for geriatric femoral neck fractures compared to traditional cannulated screw fixation methods. To assess the clinical efficacy of these two internal fixation methods in the treatment of geriatric femoral neck fractures, this study selected 175 patients treated at our hospital between January 2022 and December 2022 as the research objects. Based on the different internal fixation methods used, the patients were divided into a control group and an observation group. The clinical efficacy of locking compression plate fixation vs. cannulated screw fixation for geriatric femoral neck fractures was compared. The results showed that there were no statistically significant differences in surgical time, incision length, intraoperative blood loss, postoperative hemoglobin decrease, incision healing grade, fracture healing time, and Harris scores between the two groups. The time to initiate partial weight-bearing exercises was significantly earlier in the observation group compared to the control group (*P* < 0.05), suggesting that locking compression plate fixation offers an advantage in early postoperative functional recovery. Further analysis revealed that the incidence of postoperative complications was significantly lower in the observation group than in the control group (*P* < 0.05), which may be attributed to the locking compression plate, with its fixed-angle screw-plate construct, forms an internal support structure that provides superior axial, torsional, and lateral stability compared to the point-contact fixation of Cannulated Screw. This enhanced biomechanical stability effectively reduces micromotion and shear forces at the fracture site, thereby lowering the incidence of complications such as delayed wound healing and implant loosening. In addition, the range of motion (ROM) for hip flexion and extension at 1 and 6 months postoperatively was significantly better in the observation group, and the ROM for hip internal and external rotation at 1 month was also markedly higher (*P* < 0.01), indicating that locking compression plate fixation facilitates improved joint function recovery. This functional advantage may be related to the enhanced three-dimensional stability and the feasibility of early postoperative rehabilitation. Moreover, the observation group demonstrated significantly lower VAS pain scores at 1 month postoperatively compared to the control group (*P* < 0.01), indicating a superior effect in alleviating postoperative pain.

This study has several limitations. First, as a retrospective study, it may be subject to selection bias and information bias in terms of patient enrollment, data collection, and variable control, making it difficult to completely eliminate the influence of confounding factors and thus limiting the ability to draw causal inferences. In contrast, prospective randomized controlled trials can more effectively control for such confounders and provide a higher level of evidence; future studies of this type are needed to further validate the reliability of our findings. Second, the relatively small sample size may reduce the statistical power of some of the conclusions. Third, important factors affecting postoperative recovery—such as the degree of osteoporosis, nutritional status, and patient compliance—were not fully quantified in this study. In addition, postoperative functional assessments were mainly focused on short-term follow-up time points, and long-term outcomes and quality of life indicators were not evaluated, which limits the generalizability of the conclusions. Therefore, future research should involve larger sample sizes, prospective study designs, and extended follow-up periods to further investigate the efficacy of different internal fixation methods in elderly patients with femoral neck fractures. At the same time, this manuscript lacks illustrative diagrams, which makes the data less intuitive and harder to interpret.

In conclusion, in terms of the clinical treatment of geriatric femoral neck fractures, internal fixation methods can be employed. Compared to cannulated screw fixation, locking compression plate fixation can achieve better clinical outcomes. It allows patients to engage in early functional recovery training after getting out of bed, reducing the occurrence of postoperative complications. This provides a certain basis for the promotion of locking compression plate fixation in clinical practice.

## Data Availability

The raw data supporting the conclusions of this article will be made available by the authors, without undue reservation.
